# Novel Therapies for Tongue Squamous Cell Carcinoma Patients with High-Grade Tumors

**DOI:** 10.3390/life11080813

**Published:** 2021-08-10

**Authors:** Yinghua Li, Hao Lin, Lu Chen, Zihao Chen, Weizhong Li

**Affiliations:** 1 Department of Oral and Maxillofacial Surgery, Nanfang Hospital, Southern Medical University, Guangzhou 510515, China; liyinghua0611@outlook.com; 2 Department of Urology, Nanfang Hospital, Southern Medical University, Guangzhou 510515, China; linhao12@smu.edu.cn; 3 School of Clinical Medicine, Baotou Medical College, Baotou 014040, China; chenludr@gmail.com

**Keywords:** TSCC, cell cycle, immunotherapy, drug candidates

## Abstract

Background: Tongue squamous cell carcinoma (TSCC) patients with high-grade tumors usually suffer from high occurrence and poor prognosis. The current study aimed at finding the biomarkers related to tumor grades and proposing potential therapies by these biomarkers. Methods: The mRNA expression matrix of TSCC samples from The Cancer Genome Atlas (TCGA) database was analyzed to identify hub proteins related to tumor grades. The mRNA expression patterns of these hub proteins between TSCC and adjacent control samples were validated in three independent TSCC data sets (i.e., GSE9844, GSE30784, and GSE13601). The correlation between cell cycle index and immunotherapy efficacy was tested on the IMvigor210 data set. Based on the structure of hub proteins, virtual screening was applied to compounds to find the potential inhibitors. Results: A total of six cell cycle biomarkers (i.e., BUB1, CCNB2, CDC6, CDC20, CDK1, and MCM2) were selected as hub proteins by protein–protein interaction (PPI) analysis. In the validation data sets, the mRNA expression levels of these hub proteins were higher in tumor samples versus normal controls. The cell cycle index was constructed by the mRNA expression levels of these hub proteins, and patients with a high cell cycle index demonstrated favorable drug response to the immunotherapy. Three small molecules (i.e., ZINC100052685, ZINC8214703, and ZINC85537014) were found to bind with hub proteins and selected as drug candidates. Conclusion: The cell cycle index might provide a novel reference for selecting appropriate cancer patient candidates for immunotherapy. The current research might contribute to the development of precision medicine and improve the prognosis of TSCC.

## 1. Introduction

Oral squamous cell carcinoma (OSCC) is one of the most common malignant diseases, and it has a poor prognosis since more than 40% of patients do not survive more than 5 years [1]. It caused 377,713 cases and 177,757 deaths in 2020 [2]. Some risk factors, such as smoking, alcohol addiction, betel quid chewing, have positive correlations with the occurrence of OSCC [3]. Tongue squamous cell carcinoma (TSCC) is the most common subtype of OSCC, since it occupies more than 40% of the OSCC samples and has a worse prognosis [4]. There are some disadvantages in prevalent treatment approaches such as surgical resection, chemotherapies, and radiotherapies [5]. Firstly, the survival rate of TSCC patients is still low, even after these therapies [6]. Secondly, these prevalent therapies could lead to disfigurement or functional impairment, for example, surgical resection could cause permanent disfigurement, and chemotherapies and radiotherapies are associated with toxicities [5]. Therefore, novel therapies are necessary for TSCC patients.

Recently, immunotherapies, such as PD-1 and PD-L1 antibodies, have been tested in a range of clinical trials and have obtained great success [7]. In 2016, nivolumab, a monoclonal antibody targeting PD-1, was approved by the FDA for the treatment of metastatic squamous cell cancer of head and neck [8]. TSCC samples were found to be correlated with high PD-1/PD-L1 expression and immune suppression, which indicated that TSCC could be the FDA indication of PD-1 antibody in the future [9]. PD-L1-expressing TSCC cells could evade immune surveillance, and the inhibition of PD-1/PD-L1 was proposed to prevent the initiation of carcinogenesis and treat advanced TSCC [10]. But immunotherapies have limitations and challenges because of the risk of side effects and unsatisfactory objective response rates (ORRs) [11]. For example, the occurrence rates of adverse reactions, including hypothyroidism, colitis, and pneumonitis, increased with a PD-1 antibody treatment group than a control group [12]. In addition, the response rates to PD-1 and PD-L1 antibodies were only approximately 20% in most solid tumors [13]. Therefore, the biomarkers and evaluation and selection of appropriate patients for immunotherapy are urgently needed. 

TSCC tumor grades, meaning the degree of differentiation of tumor cells, are negatively correlated with prognosis [14]. Higher grade tumors, such as grade 3 tumors, are usually used to describe the cancer cells that are poorly differentiated with lymph node metastasis [15]. Moreover, grade 3 TSCC has a worse prognosis and is more aggressive than grade 1 and 2 TSCC [15]. High-grade cancers are characterized by excessive cell cycle activity that is regulated by cell cycle markers such as cyclin-dependent kinases (CDKs) [16,17]. For example, the level of CDK2 is significantly higher in tumor cells than in normal cells [18]. Thus, novel drugs targeting cell cycle markers might contribute to improving the prognosis of TSCC patients with high-grade tumors. 

Multiple computational approaches were selected to identify the hub proteins related to tumor grades. A total of six cell cycle markers were selected as hub proteins because of their key roles in protein–protein networks. Interestingly, we found that these cell cycle markers were negatively correlated with immune cell enrichment scores. The high level of cell cycle markers indicated higher response rates and a better prognosis to immunotherapy. Cell cycle index, computed by the mRNA expression of these cell cycle markers, was proposed in the current study. Cancer patients with a high cell cycle index had a worse prognosis in prevalent therapies but a better prognosis in immunotherapy. Furthermore, a molecular docking approach was used to identify drug candidates targeting cell cycle markers for patients with high tumor grades. In summary, our study proposed potential therapies such as immunotherapy and compounds for TSCC patients with high-grade tumors.

## 2. Material and Methods

### 2.1. Data Processing and Differential Expression Analysis

In the current study, 3 groups, including 5 different data sets, were downloaded and analyzed. (1) The data set in the first group was a TCGA–TSCC dataset. The RNA sequencing data profiles and clinical information of 139 TSCC samples (126 cancer and 13 normal samples) were downloaded from TCGA. The data set in the first group was used to determine the hub proteins. (2) The data sets in the second group were GSE9844 (26 TSCC and 12 normal samples) [19], GSE30784 (184 TSCC and 45 normal samples) [20], and GSE13601 (26 TSCC and 32 normal samples) [21]. The expression matrix of each data set was downloaded from the Gene Expression Omnibus (GEO). The data sets in the second group were used to validate the expression pattern of hub proteins. (3) The dataset in the third group was IMvigor210 cohort. The gene expression matrix and relevant clinical follow-up records of the IMvigor210 cohort (348 cancer patients treated with atezolizumab/anti-PD-L1) were obtained from the previous article [22]. The data set in the third group was used to validate the impact of the cell cycle index on immunotherapy efficacy. 

In order to select the genes that are crucial for the tumor occurrence, the differentially expressed genes (DEGs) between the tumor and adjacent controls from TCGA–TSCC data set were chosen by “edgeR” package in the R language [23]. The values of fold change and *p*-value were calculated for each gene, and the genes with a log_2_FoldChange more than 1 and a *p*-value less than 0.05 were selected as DEGs.

### 2.2. Selection of Grade-Associated Genes

To identify the hub genes/proteins for tumor grades, the mRNA expression levels of DEGs were chosen to determine the genes that correlated with tumor grade. The selection process contained two steps: (1) DEGs were chosen by differential expression analysis among tumor grades using the Kruskal–Wallis rank-sum test (*p*-value < 0.05). (2) DEGs were chosen by Pearson correlation analysis (*p*-value < 0.05). The filtered DEGs were defined as grade-associated genes.

### 2.3. Functional Enrichment Analysis 

Gene Ontology (GO) and pathway enrichment analysis were applied using the “Clusterprofiler” package in the R language [21]. The input data for this package were the gene symbols of grade-associated genes, and the output were the enriched GO terms and Kyoto Encyclopedia of Genes and Genomes (KEGG) pathways. The significantly enriched terms and pathways were selected by the criterion: *p*-value < 0.05.

### 2.4. Identification of Survival Analysis of Hub Genes

Protein–protein interaction (PPI) networks of the grade-associated genes were constructed to select the hub genes/proteins. All grade-associated genes were uploaded to the STRING website (https://string-db.org/) (accessed on 31 June 2020), and only the interactions that came from experiments were retained. Moreover, the interactions were defined as significant interactions when their confidence value were more than 0.7. After obtaining the PPI network, the genes/proteins were characterized as hub genes/proteins when their “Degree” value was more than 4. The correlations between hub genes and prognosis of TSCC were calculated by the survival analysis. 

### 2.5. Enrichment Scores of Immune Cell Types 

Based on the expression data of TSCC samples and existing immune-related gene signatures, an existing method, namely, the Single Sample Gene Set Enrichment Analysis (ssGSEA) method, was used to calculate the immune cell enrichment scores of immune cell types. The ssGSEA, derived from the GSEA method, can be implemented using the “GSVA” package in the R language [24]. The enrichment scores were used to reflect the infiltration degree of immune cells within a sample.

### 2.6. Structure-Based Virtual Screening

The virtual screening approach was used to select novel drug candidates for targeting hub proteins. The structures of receptors (proteins) and ligands (small molecules) were necessary input data for virtual screening. The most crucial parameter in the process of virtual screening was the binding sites. The 3D structures of receptors and the targeting sites in the virtual screening were from the Protein Data Bank (PDB) data set. On the other hand, the structures of ligands were downloaded from the ZINC15 database. Virtual screening was carried out using the LibDock module of Discovery Studio. LibDock [25] is a rigid-based docking module, and it consists of: (1) generating small-molecule conformations; (2) obtaining the target site of proteins (hot spot); (3) matching the target site and the small molecule; (4) scoring the binding poses. 

### 2.7. ADMET (Adsorption, Distribution, Metabolism, Excretion, and Toxicity) Prediction

The quality control of drugs, such as ADMET, directly determines the potential clinical application, and we predicted ADMET via in silicon methods. The pharmacokinetics indicators, such as aqueous solubility and human intestinal absorption of all small molecule drugs, were assessed by ADME application of Discovery Studio. To investigate the toxic effect of these small molecule drugs, TOPKAT application from Discovery Studio was used to predict the rodent carcinogenicity, developmental toxicity potential (DTP) properties, and Ames mutagenicity. 

### 2.8. Statistical Analysis and Ethics Statement

For survival analysis, the Kaplan–Meier method from the package “survival” of the R language was selected to compare overall survival (OS) time among different groups [26]. For each hub gene, the mean values of tumor and adjacent control cohorts were compared using the Wilcoxon rank-sum test. All mRNA expression data profiles, corresponding clinical information, and 3D structures of hub proteins were retrieved from publicly available data sets, which are free to download and analyze without limitations. Investigators of each study obtained approval from their local ethics committee and informed consent from patients.

## 3. Results

### 3.1. Identification of DEGs and Grade-Associated Genes

**Figure 1:**
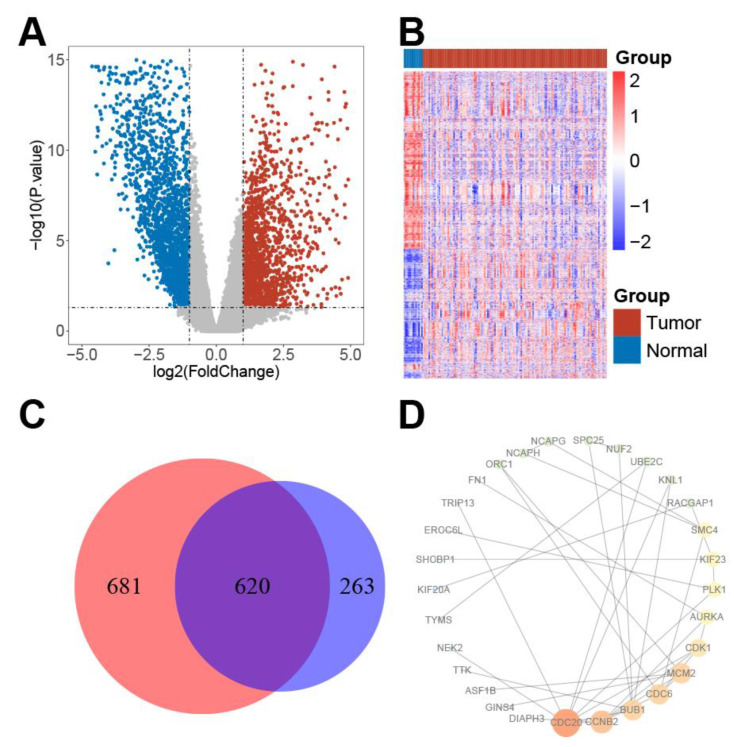
DEG analysis: (**A**) Volcano plot visualizing the DEGs. The vertical lines demarcate the log_2_FoldChange, values while the horizontal line marks a –log_10_*p*-value of 0.05. Red represents the upregulated genes, while green represents the downregulated genes. (**B**) Heatmap of the DEGs. The right longitudinal axis shows the clustering information of the samples. The samples were mainly divided into two major clusters, and these two clusters were the tumor tissue and adjacent normal tissue. (**C**) 883 and 1301 genes were found to be significant in Kruskal–Wallis rank-sum test and Pearson correlation coefficient analysis, respectively. A total of 620 genes were defined as grade-associated genes. (**D**) Protein–protein interaction network of grade-associated genes. The color intensity and the size of nodes were positively correlated with the degree score. Abbreviations: DEG, differently expressed genes; TSCC, tongue squamous cell carcinoma; TCGA, The Cancer Genome Atlas.

A total of 126 TSCC samples and 13 normal controls were downloaded from the TCGA data set. A total of 3728 genes/mRNAs (1526 over-expressed and 2202 down expressed genes) were selected by the cut-off of *p*-value < 0.05 and log_2_FoldChange > 1 (Figure 1A,B). 

The Kruskal–Wallis rank-sum test and Pearson correlation coefficient analysis were used to find potential drug targets that were differentially expressed among tumor grades and positively correlated with tumor grades. Eight hundred and eighty-three and 1301 genes were found to be significant in the Kruskal–Wallis rank-sum test and Pearson correlation coefficient analysis, respectively (Figure 1C). A total of 620 overlapping genes were defined as grade-associated genes (Figure 1C). 

### 3.2. Enrichment Analysis on Grade-Associated Genes

All 620 grade-associated genes were selected to identify significantly enriched GO terms and KEGG pathways (Table 1). Grade-associated genes were involved in the different GO terms: (1) biological process (BP): organelle fission, nuclear division, and chromosome degradation; (2) cellular component (CC): extracellular matrix, spindle, and collagen-containing extracellular matrix; (3) molecular function (MF): cofactor binding, actin binding, and coenzyme binding. Grade-associated genes were also associated with KEGG pathways, such as cell cycle, regulation of actin cytoskeleton, and focal adhesion. 

### 3.3. Protein–Protein Interaction (PPI) and Hub Proteins

**Figure 2:**
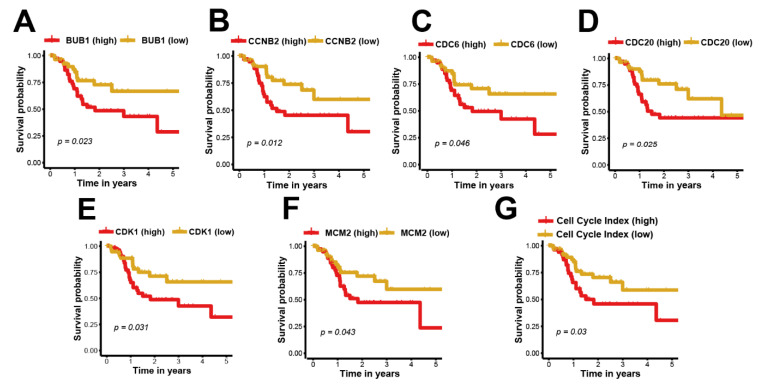
Survival analysis of hub proteins and cell cycle index. (**A**) Survival analysis of the association between the BUB1 expression and overall survival time in the TCGA–TSCC data set. (**B**) Survival analysis of the association between the CCNB2 expression and overall survival time in the TCGA–TSCC data set. (**C**) Survival analysis of the association between the CDC6 expression and overall survival time in the TCGA–TSCC data set. (**D**) Survival analysis of the association between the CDC20 expression and overall survival time in the TCGA–TSCC data set. (**E**) Survival analysis of the association between the CDK1 expression and overall survival time in the TCGA–TSCC data set. (**F**) Survival analysis of the association between the MCM2 expression and overall survival time in the TCGA–TSCC data set. (**G**) Survival analysis of the association between the cell cycle index and overall survival time in the TCGA–TSCC data set. The Kaplan–Meier survival curve revealed that high BUB1, CCNB2, CDC6, CDC20, CDK1, and MCM2 expression and the cell cycle index conferred the worst overall survival in patients with TSCC (*p* < 0.05).

In Figure 1D, a PPI network was formed by the proteins from grade-associated genes. In the network, a protein that did not have an interaction with other proteins was removed. Six hub proteins (i.e., BUB1, CCNB2, CDC6, CDC20, CDK1, and MCM2) were characterized as hub proteins since their “degree” values were more than 10, which indicated that they played core roles in the regulatory network. In addition, all six proteins were found to be enriched in the KEGG pathway of the cell cycle (Table 1). Moreover, survival analysis results in TSCC patients from the TCGA data set demonstrated that these hub proteins had a negative effect on the prognosis (Figure 2A,F). In the TSCC samples from the TCGA dataset, the mRNA expression levels of all six hub proteins were significantly higher in cancer patients than in controls (Table 2). In order to evaluate the degree of the cell cycle of the TSCC tumors, we proposed a cell cycle index by these six hub proteins. In the present study, the cell cycle index was calculated by the sum of mRNA expression levels of six hub proteins. The survival result demonstrated that the cell cycle index was a significantly negative prognostic factor for survival time of TSCC patients (Figure 2G).

### 3.4. Validation of Hub Genes in Independent Data Sets

Six hub proteins, including budding uninhibited by benzimidazoles 1 (BUB1), cyclin B2 (CCNB2), cell division cycle 6 (CDC6), cell division cycle 20 (CDC20), cyclin dependent kinase 1 (CDK1), and minichromosome maintenance complex component 2 (MCM2) were found to be positively correlated with tumor grades of patients from the TCGA–TSCC data set (Supplementary Materials, Figure S1A–F). In Table 2, the expression patterns of these hub genes were validated using three independent TSCC data sets. In GSE30784, tumor samples expressed higher levels of all six hub genes. In GSE9844, the expression levels of five hub genes were found to be higher in tumor tissues. In GSE13601, five of six hub genes had higher expression levels in tumor tissues.

### 3.5. Correlation of Hub Genes with Immune Cell Enrichment Scores

**Figure 3:**
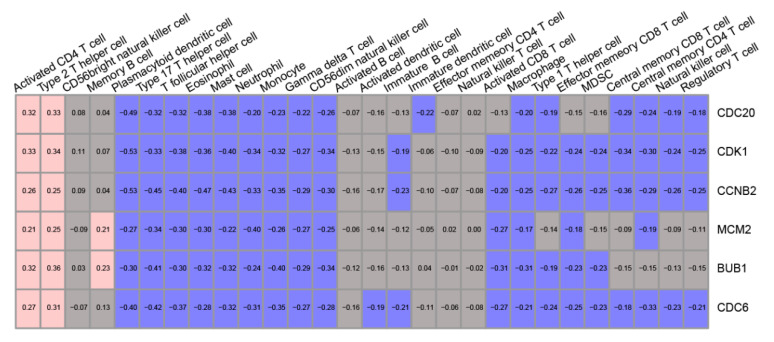
The correlations of immune cell enrichment scores and hub gene expression levels. Blue: negative correlation, gray: nonsignificant correlation, red: positive correlation.

**Figure 4:**
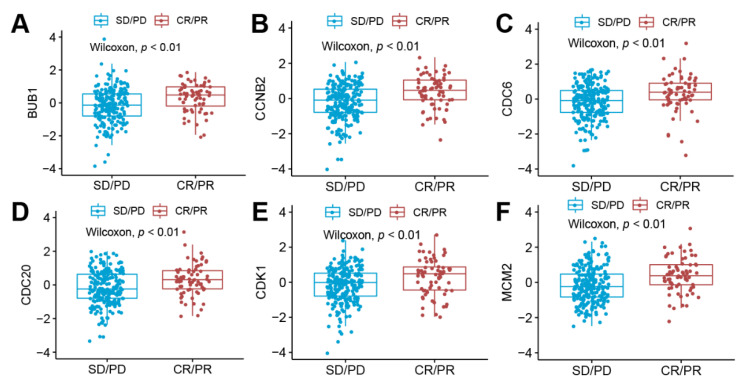
The correlation between the mRNA expression levels of hub proteins with the drug response to immunotherapy (atezolizumab/anti-PD-L1) in the validation data set (Imvigor210). (**A**) The box plot revealed that high BUB1 expression was associated with the better response to immunotherapy (*p* < 0.05). (**B**) The box plot revealed that high CCNB2 expression was associated with the better response to immunotherapy (*p* < 0.05). (**C**) The box plot revealed that high CDC6 expression was associated with the better response to immunotherapy (*p* < 0.05). (**D**) The box plot revealed that high CDC20 expression was associated with the better response to immunotherapy (*p* < 0.05). (**E**) The box plot revealed that high CDK1 expression was associated with the better response to immunotherapy (*p* < 0.05). (**F**) The box plot revealed that high MCM2 expression was associated with the better response to immunotherapy (*p* < 0.05)**.** Abbreviation: CR, complete response; PR, partial response; SD, stable disease; PD, progressive disease.

**Figure 5:**
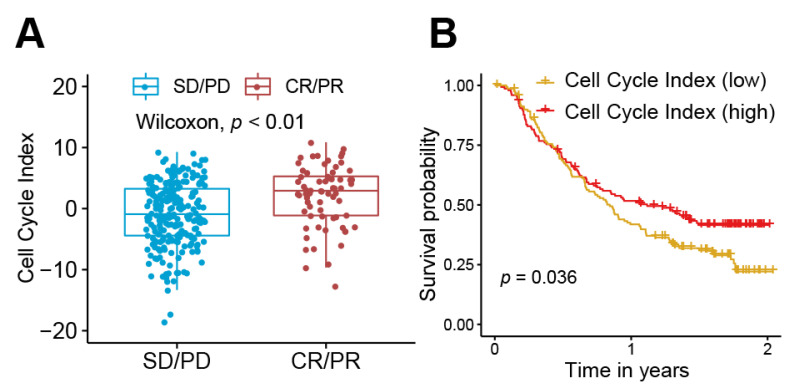
Validation of the cell cycle index (sum of the expression levels of hub genes). (**A**) The response to immunotherapy (atezolizumab/anti-PD-L1) was related to high expression of the cell cycle index. (**B**) The patients with high a cell cycle index were correlated with a better prognosis. Abbreviation: CR, complete response; PR, partial response; SD, stable disease; PD, progressive disease.

These hub genes were also found to be significantly correlated with immune cell enrichment scores (Figure 3). For activated CD4 T cells and Type 2 helper cell, the associations are positive. On the contrary, a variety of immune cells, such as dendritic cells, neutrophils, and CD8 T cells, exhibited a significant negative correlation with these hub genes. The impact of these hub genes on the results of treatment and prognosis of immune checkpoint inhibitors (atezolizumab/anti-PD-L1) were validated in the independent data set. The expression levels of these six hub genes were higher in the drug-responsive group than the drug-non-responsive group (Figure 4). Patients with high BUB1 and MCM2 expression levels showed a better prognosis than the low expression group (Supplementary Materials, Figure S2A–F). The high level of cell cycle index was correlated with higher efficacy (Figure 5A) and better prognosis (Figure 5B) of atezolizumab/anti-PD-L1. These results were opposite to the survival results from the TCGA–TSCC cohort (Figure 2).

### 3.6. Virtual Screening of Compounds

**Figure 6:**
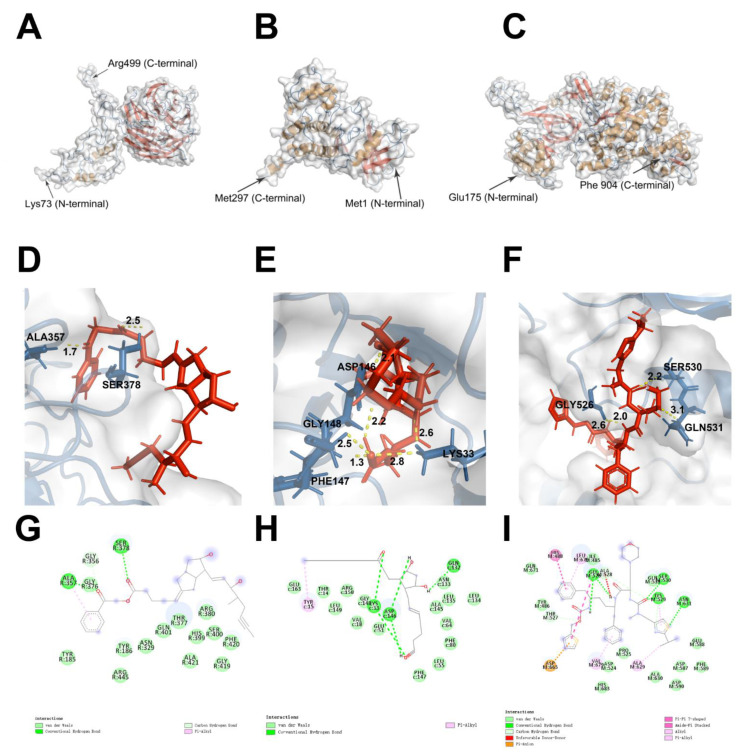
3D structures of hub proteins (**A**) CDC20, (**B**) CDK1, and (**C**) MCM2. In the structure of each protein, secondary structures were represented by colors (β-sheets: red; coil: blue; α helix: brown). The docking structure of hub proteins and the compounds: (**D**) ZINC100052685 with CDC20, (**E**) ZINC8214703 with CDK1, and (**F**) ZINC85537014 with MCM2. The proteins, compounds, and hydrogen bonds were colored by blue, red, and yellow colors, respectively. Schematic of intermolecular interaction of the predicted binding modes of (**G**) ZINC100052685 with CDC20, (**H**) ZINC8214703 with CDK1, and (**I**) ZINC85537014 with MCM2.

To select the small molecules that could interact with these hub proteins, the virtual screening method was used. Among these six hub proteins, the structure of CDC20, CDK1, and MCM2 were downloaded from Protein Data Bank (PDB) dataset (Figure 6A-C). The binding sites of these hub proteins were obtained from their PDB files and defined as SER377:HIS380 (CDC20), ALA145:PHE147 (CDK1), and PRO525:GLN531 (MCM2). The structure-based virtual screening using Libdock application of discovery studio was performed on 1615 FDA-approved small molecule drugs. A total of 541, 841, and 1591 small molecule drugs had high binding affinities to CDC20, CDK1, and MCM2, respectively. 9 drugs were exhibited in Table 3 since they had the highest Libdock score.

### 3.7. Pharmacologic Properties of Compounds

The Pharmacologic properties and toxicity results of all small-molecule drugs are provided in Table 4 and Table 5: (1) nine small molecules were soluble; (2) four small-molecules CYP2D6 inhibitors; (3) three small molecules were toxic in hepatotoxicity; (4) six small molecules had a good absorption level; (5) three small molecules (i.e., ZINC100052685, ZINC8214703, and ZINC85537014) were predicted to be non-toxic in developmental potential. Therefore, ZINC100052685, ZINC8214703, and ZINC85537014 were selected as safe compounds. Two, 6, and 4 hydrogen bonds were found in the ZINC100052685-CDC20 (Figure 6D), ZINC8214703-CDK1 (Figure 6E), and ZINC85537014-MCM2 (Figure 6F) complex. Fourteen, 15, and 14 van der Waals interactions were found in the ZINC100052685-CDC20 (Figure 6G), ZINC8214703-CDK1 (Figure 6H), and ZINC85537014-MCM2 (Figure 6I) complex.

## 4. Discussion

Although recent advances in therapeutic strategies, such as surgical treatment and chemotherapy, are applied, the mortality of TSCC patients with high-grade tumors is still relatively high [6]. In the present study, computational methods were used to select six hub proteins positively associated with tumor grade. After validation in three independent data sets, these six hub proteins were found to be robustly and highly expressed in TSCC samples. Patients with a high cell cycle index (sum of mRNA levels of six hub proteins) had a tendency to be more responsive to immunotherapy. Structure-based virtual screening was used to identify the compounds for targeting the hub protein. Thus, our study revealed the biomarkers related to tumor grades and proposed potential therapies such as immunotherapy and compounds for TSCC patients with high-grade tumors using these biomarkers. 

In the current study, six hub proteins screened in the TCGA–TSCC data set were then verified using the three independent TSCC GEO data sets. We further analyzed the effect of these six hub genes on the prognosis of TSCC patients and their interaction networks. These six hub proteins were found to be enriched in the cell cycle pathway. *BUB1* has a crucial role in mitosis [27], and over-expressed *BUB1* contributes to tumor formation [28]. *CCNB2* is one of the essential components of the cell cycle regulatory machinery [29]. Cell division cycle 6 (CDC6) overexpression promotes DNA hyper-replication [30]. Knockdown of CDC6 slowed cancer cell growth, altered cell cycle progression, and inhibited cell proliferation [31]. Tongue cancer patients with high expression of CDC20 demonstrated a worse prognosis [32], and CDC20 inhibitors were thought to be promising strategies for cancers [33]. A high level of CDK1 expression was positively associated with tumor grades of tongue cancer and negatively correlated with the survival time [34]. The levels of *MCM2* mRNA were significantly elevated in tongue cancer compared to normal controls [35]. In addition, the reduced expression of MCM2 could improve the drug response of chemical drugs [36]. 

The high expression of PD-1 and its cognate ligands PD-L1/PD-L2 could suppress T cell activation/proliferation [37]. Cancer cells could cause overstimulation of the PD-1/PD-L1 signaling pathway to suppress T cell activation/proliferation and antigen-specific T cell immune response, thereby bypassing immune surveillance and enhancing cancer cell survival [38]. PD-1/PD-L1 antibodies could block this pathway, increase immune cell proliferation, and enhance the efficacy of the body’s natural antitumor surveillance system [38]. Six PD-1/PD-L1 antibodies have been approved with supplemental indications across 19 cancer types and two tissue-agnostic conditions [37]. However, the response rate to PD-1 and PD-L1 antibodies was only approximately 20% in most solid tumors. Several biomarkers, such as tumor mutational burden (TMB) and the level of PD-1/PD-L1, have been proposed to be predictors of immunotherapy outcomes. However, there are some disadvantages to these biomarkers. For example, the expression level of PD-L1 is unstable because it can be affected by mTOR inhibitors. The precise TMB result is only originated from whole-exome sequencing results that require more time and money [39]. In order to solve this problem, some articles provided to identify different immune subtypes by these prevalent biomarkers [40]. Epithelial-to-mesenchymal transition (EMT) is characterized by the epithelial dedifferentiation to the mesenchymal phenotype [41]. The expression levels of EMT-related genes are correlated with lower response rates and worse prognosis to immunotherapy [42]. However, the disadvantages of these prevalent biomarkers suggested that novel robust biomarkers are needed. A previous study found that 10 cell proliferation genes, including BUB1, CCNB2, and CDK1, could act as indicators for response to immune checkpoint inhibitors in PD-L1 negative renal cell carcinoma [43]. In the present study, the six hub proteins were found to be negatively correlated with most immune cell types such as CD4 T cell, B cell, and CD8 T cell (Figure 3). These results suggest that the T cell activation/proliferation is suppressed in the sample with high expression of six hub proteins. Another study also found that cell cycle biomarkers, such as CDK1 and CCNB2, were positively correlated with the levels of immunotherapy indicators such as PD-1, PDL-1, and CTLA-4 [44]. Therefore, the six hub proteins might contribute to the T-cell exhaustion and tumor survival via regulating PD-1/PD-L1 signaling [44], and this regulation could be blocked in the treatment of PD-1/PDL-1 antibodies. However, further research into these mechanisms is required.

Although the six hub proteins and cell cycle index had negative impacts on the prognosis of cancer patients with the treatment of chemotherapy and surgery (Figure 2), they were positively associated with drug response and prognosis of cancer patients with the treatment of immunotherapy (Figure 4 and Figure 5). These results suggest that the six hub proteins and cell cycle index could be used as biomarkers for the selection of appropriate cancer patient candidates for receiving immunotherapy. The patients with high expression levels of the six hub proteins and cell cycle index are more likely to have a drug response to PD-1/PD-L1 antibodies. The expression levels of these six hub proteins could be detected before the clinical application of PD-1/PD-L1 antibodies. These results and conclusions are crucial for indicating the use of immunotherapy on TSCC patients and also contribute to the prognosis of patients with a higher cell cycle index.

We also selected the potential small molecule drugs targeting cell cycle index proteins. Three small-molecule drugs (i.e., ZINC100052685, ZINC8214703, and ZINC85537014) could firmly bind to the active site cavity of the cell cycle hub proteins. The results of pharmacological properties also indicated that they had the potential to act as anti-tumor agents. ZINC100052685, namely, iloprost, is capable of affecting platelet aggregation and treating pulmonary arterial hypertension [45]. Iloprost could reduce the expression of matrix metallopeptidase-2 and then attenuate ovarian cancer progression [46]. Iloprost could also improve endobronchial dysplasia and prevent the development of lung cancer. ZINC8214703, namely, unoprostone, could reduce intraocular pressure and treat glaucoma [47]. ZINC85537014, namely, cobicistat, has the ability to treat human immunodeficiency virus (HIV) infection [48] and increase the response rates of anticancer drugs [49]. Because of the anticancer properties of iloprost and unoprostone, these two small molecules might be new drug candidates for TSCC patients [50]. In conclusion, ZINC100052685, ZINC8214703, and ZINC85537014 could be promising drug candidates for patients with high-grade TSCC tumors. However, these results need more experimental studies to validate. 

## 5. Conclusions

The present study identified six hub proteins related to tumor grades, and we constructed the cell cycle index by mRNA expression levels of these hub proteins. Our research found that patients with higher a cell cycle index had a better prognosis after the treatment of immunotherapy. In addition, we found that the three compounds could firmly target these hub proteins. In summary, this research might contribute to providing novel therapies and improving the prognosis of TSCC patients with high-grade tumors. 

## Figures and Tables

**Table 1:** Functional and pathway enrichment analysis of grade-associated genes in TSCC.

ID	Term	*p*-Value	Count
KEGG:hsa04110	Cell Cycle	<0.01	15
KEGG:hsa04810	Regulation of Actin Cytoskeleton	<0.01	14
KEGG:hsa04510	Focal Adhesion	<0.01	13
KEGG:hsa04512	ECM-Receptor Interaction	<0.01	11
KEGG:hsa04914	Progesterone-mediated Oocyte Maturation	<0.01	11
GO_BP:0048285	Organelle Fission	<0.01	48
GO_BP:0000280	Nuclear Division	<0.01	47
GO_BP:0007059	Chromosome Segregation	<0.01	45
GO_BP:0140014	Mitotic Nuclear Division	<0.01	42
GO_BP:0043062	Extracellular Structure Organization	<0.01	40
GO_CC:0031012	Extracellular Matrix	<0.01	41
GO_CC:0005819	Spindle	<0.01	38
GO_CC:0062023	Collagen-containing Extracellular Matrix	<0.01	38
GO_CC:0098687	Chromosomal Region	<0.01	33
GO_CC:0000775	Chromosome, Centromeric Region	<0.01	28
GO_MF:0048037	Cofactor Binding	<0.01	28
GO_MF:0003779	Actin Binding	<0.01	24
GO_MF:0050662	Coenzyme Binding	<0.01	22
GO_MF:0005201	Extracellular Matrix Structural Constituent	<0.01	19
GO_MF:1901681	Sulfur Compound Binding	<0.01	18

The top five terms were selected according to the *p*-value and the gene count number. Abbreviations: TSCC, tongue squamous cell carcinoma; KEGG, Kyoto Encyclopedia of Genes and Genomes; GO, Gene Ontology; BP, biological process; CC, cellular component; MF, molecular function.

**Table 2:** Comparing mRNA expression levels of hub proteins between tumor and normal samples groups by Wilcoxon test.

Datasets\Genes	BUB1	CCNB2	CDC6	CDC20	CDK1	MCM2
TSCC (TCGA)	*p* < 0.01	*p* < 0.01	*p* < 0.01	*p* < 0.01	*p* < 0.01	*p* < 0.01
GSE30784	*p* < 0.01	*p* < 0.01	*p* < 0.01	*p* < 0.01	*p* < 0.01	*p* < 0.01
GSE9844	0.23	0.019	*p* < 0.01	*p* < 0.01	0.033	*p* < 0.01
GSE13601	*p* < 0.01	*p* < 0.01	0.14	*p* < 0.01	*p* < 0.01	*p* < 0.01

**Table 3:** The compounds with the highest Libdock score.

Protein	ZincID	Libdock Score
CDC20	ZINC3799072	116.1
CDC20	ZINC100052685	105.9
CDC20	ZINC3785268	105.6
CDK1	ZINC8214703	126.7
CDK1	ZINC21297660	113.4
CDK1	ZINC601250	111.8
MCM2	ZINC28232755	171.2
MCM2	ZINC85537014	166.8
MCM2	ZINC28639340	161.7

**Table 4:** Adsorption, distribution, metabolism, and excretion properties of compounds.

Compounds	Solubility Level	BBB Level	CYP2D6	Hepatotoxicity	Absorption Level	PPB Level
ZINC3799072	3	2	1	0	0	1
ZINC100052685	2	4	0	0	1	1
ZINC3785268	3	2	1	0	0	1
ZINC8214703	3	4	0	0	0	1
ZINC21297660	3	3	0	0	0	1
ZINC601250	1	0	1	1	1	1
ZINC28232755	1	4	0	0	3	0
ZINC85537014	3	4	1	1	2	0
ZINC28639340	3	4	0	1	2	1

Abbreviation: BBB, blood–brain barrier; CYP2D6, cytochrome P-450 2D6; PPB, plasma protein binding. Aqueous-solubility level: 0, extremely low; 1, very low, but possible; 2, low; 3, good. BBB level: 0, very high penetrant; 1, high; 2, medium; 3, low; 4, undefined. CYP2D6 level: 0, noninhibitor; 1, inhibitor. Hepatotoxicity: 0, nontoxic; 1, toxic. Human-intestinal absorption level: 0, good; 1, moderate; 2, poor; 3, very poor. PPB: 0, absorbent weak; 1, absorbent strong.

**Table 5:** Toxicities of compounds.

Compounds	Mouse NTP (Female)	Mouse NTP (Male)	Rat NTP (Female)	Rat NTP (Male)	Ames	DTP
ZINC3799072	0	0	0	0	0	1
ZINC100052685	0	0	0	0	0	0
ZINC3785268	0	0	0	0	0	1
ZINC8214703	0	0	0	0	0	0
ZINC21297660	0	1	0	1	0	1
ZINC601250	0	1	0	0	0	1
ZINC28232755	0	0	0	0	0	1
ZINC85537014	0	0	0	0	0	0
ZINC28639340	0	0	0	0	0	1

NTP, US National Toxicology Program; DTP, developmental toxicity potential. NTP: 0 (noncarcinogen); 1 (carcinogen). Ames: 0 (non-mutagen); 1 (mutagen). DTP: 0 (nontoxic); 1 (toxic).

## Data Availability

Publicly available datasets from The Cancer Genome Atlas (TCGA) and Gene Expression Omnibus (GEO) were analyzed in this study. This data from TCGA can be found here: https://portal.gdc.cancer.gov/ (accessed on 15 June 2021). This data from GEO can be found here: https://www.ncbi.nlm.nih.gov/geo/ (accessed on 15 June 2021). All the datasets could be downloaded without any limitations.

## Supplementary Materials

The following are available online at https://www.mdpi.com/2075-1729/11/8/813/s1, Figure S1: Expression pattern of hub genes in different tumor grades from TCGA-TSCC patients. (A–F) The results re-vealed that six hub proteins (BUB1, CCNB2, CDC6, CDC20, CDK1, and MCM2) were positively correlated with tumor grades (*p* < 0.05). Figure S2: Survival analysis of the association between the expression of hub genes and overall survival time in the validation dataset (Imvigor210). (A–F) The Kaplan-Meier survival curve revealed that high BUB1 and MCM2 expression conferred better overall survival in patients with TSCC (*p* < 0.05).

## Abbreviations

TSCC	Tongue squamous cell carcinoma
OSCC	oral squamous cell carcinoma
TCGA	The Cancer Genome Atlas
CDKs	cyclin-dependent kinases
DEGs	differentially expressed genes
PPI	protein–protein interaction
ORR	objective response rate
KEGG	Kyoto Encyclopedia of genes and Genomes
GO	Gene Ontology
BP	biological process
CC	cellular component
MF	molecular function
OS	overall survival
ssGSEA	single sample gene set enrichment analysis
PDB	Protein Data Bank
ADMET	adsorption, distribution, metabolism, excretion, and toxicity
DTP	developmental toxicity potential
BUB1	budding uninhibited by benzimidazoles 1
CCNB2	cyclin B2
CDC6	cell division cycle 6
CDC20	cell division cycle 20
CDK1	cyclin dependent kinase 1
MCM2	minichromosome maintenance complex component 2
TMB	tumor mutational burden
EMT	epithelial-to-mesenchymal transition
HIV	human immunodeficiency virus
