# Correction: Li et al. Novel Therapies for Tongue Squamous Cell Carcinoma Patients with High-Grade Tumors. *Life* 2021, *11*, 813

**DOI:** 10.3390/life13081624

**Published:** 2023-07-26

**Authors:** Yinghua Li, Hao Lin, Lu Chen, Zihao Chen, Weizhong Li

**Affiliations:** 1 Department of Oral and Maxillofacial Surgery, Nanfang Hospital, Southern Medical University, Guangzhou 510515, China; 2 Department of Urology, Nanfang Hospital, Southern Medical University, Guangzhou 510515, China; linhao12@smu.edu.cn; 3 School of Clinical Medicine, Baotou Medical College, Baotou 014040, China; chenludr@gmail.com

## Author Information Correction

1. Yinghua Li was not included as an author in the original publication [1]. With this correction, the order of affiliations has been adjusted accordingly. The corrected Author Contributions statement appears here:
  **Author Contributions:** Y.L. and H.L. performed the study and wrote the manuscript. L.C. contributed to data analysis. Z.C. and W.L. performed the technical modification and conceived the study.
2. For authors Zihao Chen and Weizhong Li, the correct order as follows: Zihao Chen and Weizhong Li.
3. The authors want to add Weizhong Li as corresponding author, and change Weizhong Li’s email address to liwz@smu.edu.cn.

## Affiliations Correction

In the published publication, there were errors regarding the affiliations 1–3. The updated affiliations should be:

^1^  Department of Oral and Maxillofacial Surgery, Nanfang Hospital, Southern Medical University, Guangzhou 510515, China
^2^  Department of Urology, Nanfang Hospital, Southern Medical University, Guangzhou 510515, China
^3^  School of Clinical Medicine, Baotou Medical College, Baotou 014040, China

The authors state that the scientific conclusions are unaffected. This correction was approved by the Academic Editor. The original publication has also been updated.
