# Supplementary material for: Novel Therapies for Tongue Squamous Cell Carcinoma Patients with High-Grade Tumors
Source: Life (Basel). 2021 Aug 10;11(8):813. doi: 10.3390/life11080813 (PMC8398384; doi:10.3390/life11080813)
Supplement: Supplementary file 1 [file life-11-00813-s001.zip › life-1281224-supplementary.pdf]

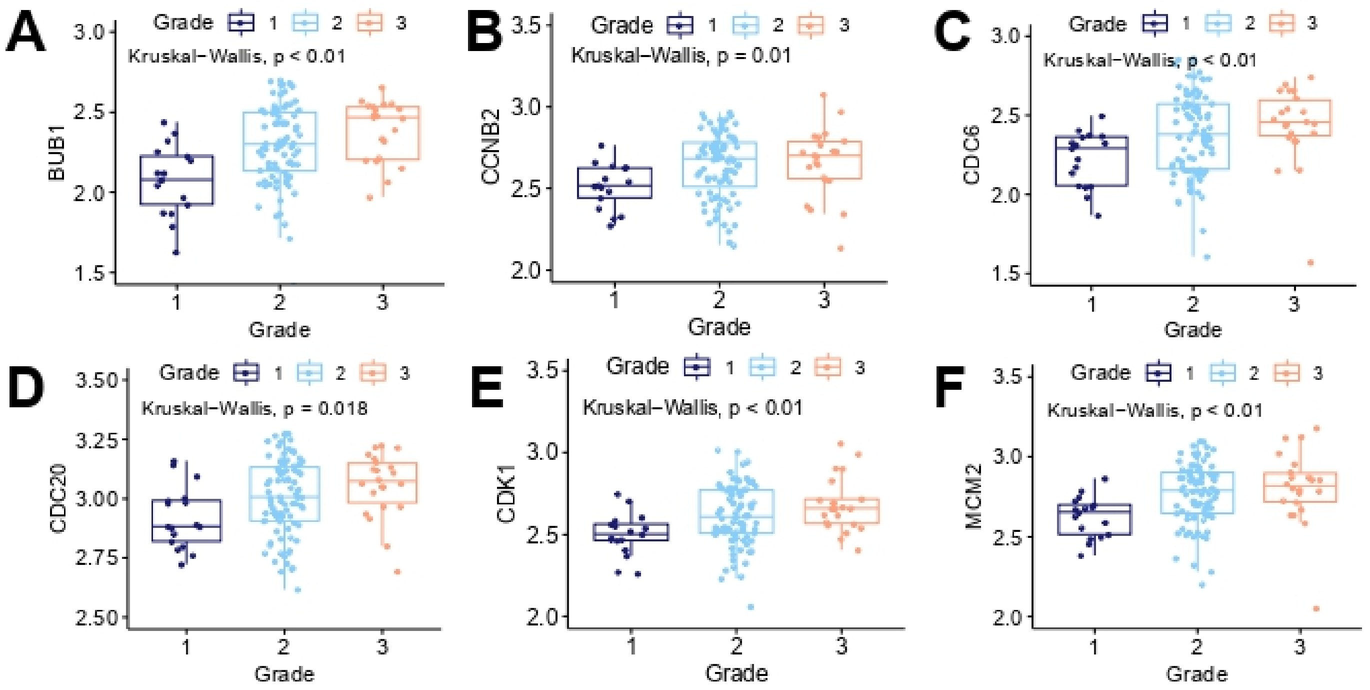

Figure S1. Expression pattern of hub genes in different tumor grades from TCGA-TSCC patients. (**a**–**f**) The results revealed that six hub proteins (BUB1, CCNB2, CDC6, CDC20, CDK1, and MCM2) were positively correlated with tumor grades ($P < 0.05$).

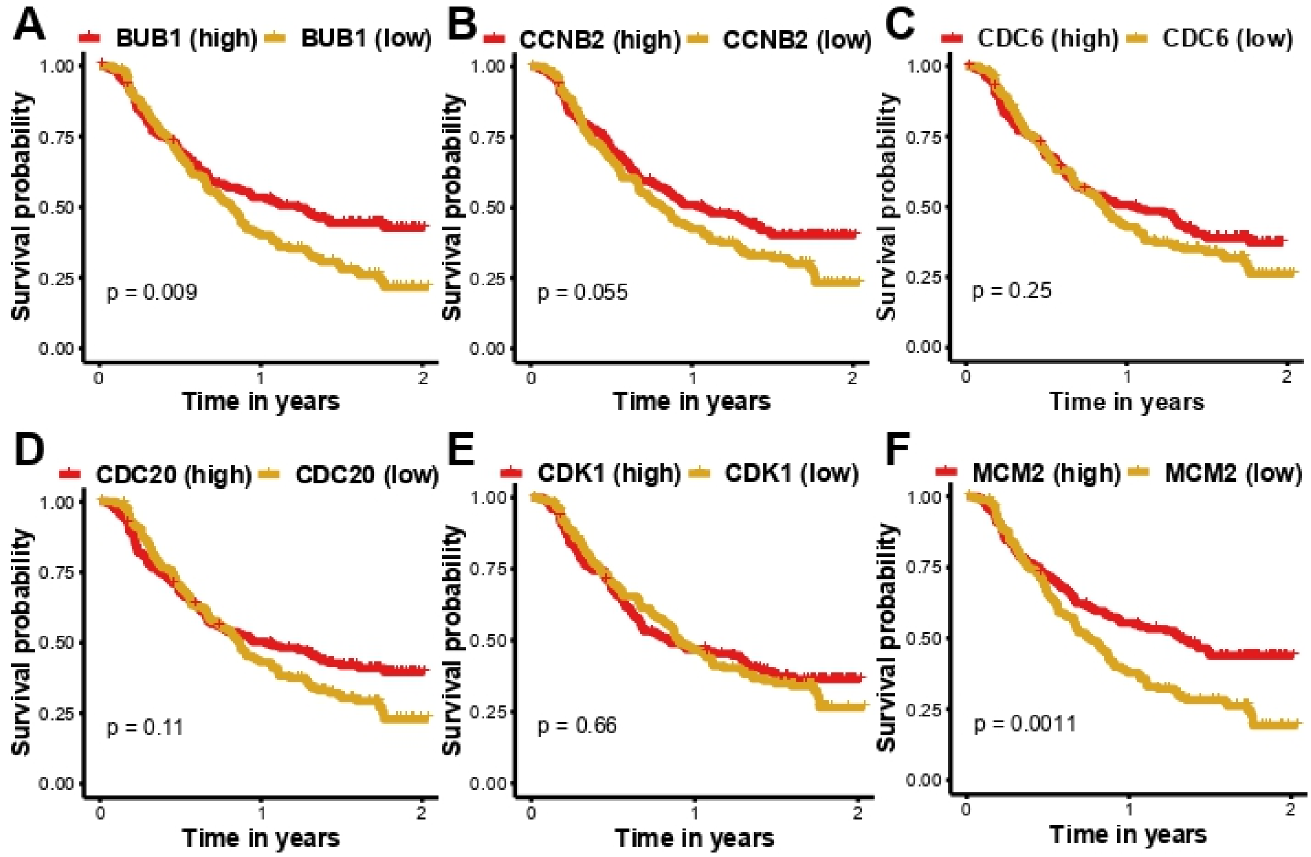

Figure 2. Survival analysis of the association between the expression of hub genes and overall survival time in the validation dataset (Imvigor210). (**a**–**f**) The Kaplan-Meier survival curve revealed that high BUB1 and MCM2 expression conferred better overall survival in patients with TSCC ($P < 0.05$).
